# The effect of tidal forcing on biogeochemical processes in intertidal salt marsh sediments

**DOI:** 10.1186/1467-4866-8-6

**Published:** 2007-06-13

**Authors:** Martial Taillefert, Stephanie Neuhuber, Gwendolyn Bristow

**Affiliations:** 1 School of Earth and Atmospheric Sciences, Georgia Institute of Technology, 311 Ferst Drive, Atlanta, GA 30332-0340, USA; 2 Department of Geodynamics and Sedimentology, University of Vienna, Althanstrasse 14, 1090 Vienna, Austria

## Abstract

**Background:**

Early diagenetic processes involved in natural organic matter (NOM) oxidation in marine sediments have been for the most part characterized after collecting sediment cores and extracting porewaters. These techniques have proven useful for deep-sea sediments where biogeochemical processes are limited to aerobic respiration, denitrification, and manganese reduction and span over several centimeters. In coastal marine sediments, however, the concentration of NOM is so high that the spatial resolution needed to characterize these processes cannot be achieved with conventional sampling techniques. In addition, coastal sediments are influenced by tidal forcing that likely affects the processes involved in carbon oxidation.

**Results:**

In this study, we used in situ voltammetry to determine the role of tidal forcing on early diagenetic processes in intertidal salt marsh sediments. We compare ex situ measurements collected seasonally, in situ profiling measurements, and in situ time series collected at several depths in the sediment during tidal cycles at two distinct stations, a small perennial creek and a mud flat. Our results indicate that the tides coupled to the salt marsh topography drastically influence the distribution of redox geochemical species and may be responsible for local differences noted year-round in the same sediments. Monitoring wells deployed to observe the effects of the tides on the vertical component of porewater transport reveal that creek sediments, because of their confinements, are exposed to much higher hydrostatic pressure gradients than mud flats.

**Conclusion:**

Our study indicates that iron reduction can be sustained in intertidal creek sediments by a combination of physical forcing and chemical oxidation, while intertidal mud flat sediments are mainly subject to sulfate reduction. These processes likely allow microbial iron reduction to be an important terminal electron accepting process in intertidal coastal sediments.

## Background

Salt marshes are coastal regions of high primary productivity [1,2] and receive substantial input of terrestrial organic matter [3]. Simultaneously, high rates of natural organic matter oxidation are recorded in salt marshes [4], and evidence suggests that secondary production in these environments depends more on estuarine primary production than terrestrially-derived organic matter [5]. In fact, these ecosystems are very often either balanced between autotrophy and heterotrophy [5] or carbon limited [4,6,7]. Salt marshes have been proposed to be a net sink of CO_2 _from the atmosphere, though remineralization of carbon exported to continental margins may in fact be a source of CO_2 _to the atmosphere [8]. To predict the relationship between the different carbon reservoirs, it is necessary to understand the processes controlling organic carbon preservation in coastal marine sediments, including salt marshes. Unfortunately, the complex interaction between physical, biological, and chemical processes in salt marsh sediments limits our ability to quantify the biogeochemical cycling of elements in these systems.

Physical (sediment transport, tidal pumping, wave action, bioturbation), chemical (oxidation-reduction, precipitation-dissolution, adsorption), and microbial processes regulate the cycling of elements in intertidal sediments. Traditionally, the high complexity of salt marsh sediment biogeochemistry has been attributed to physical mixing by bioturbation. Macroorganisms mix the sediment [9] and irrigate their burrows with dissolved oxygen [10,11]. Irrigation may also occur through the roots of *Spartina alterniflora *[12]. Since bioturbation influences biogeochemical processes, it is often considered an important forcing parameter in field studies. Bioturbation has been suggested to be responsible for the oxidation of surficial sediments [e.g., [9,13,14]], the higher rates of iron reduction measured compared to sulfate reduction [15], and the high heterogeneity of microbial populations [16].

Interestingly, biogeochemists have paid little attention to the role of tidal forcing in intertidal salt marsh sediments. These environments are characterized by complex subsurface hydrologies regulated by the marsh topography and sedimentology, influence of groundwater discharge as well as tidal pressure and wave action [17-21]. In some marshes, the subsurface flow is predominantly horizontal while, in others, water moves vertically [22]. Vertical flow can be generated at rising tide by overlying water infiltrations or water discharge to the overlying waters [22,23]. Infiltrations may either take place when water fills empty pore spaces above the water table [24], when cold overlying waters mix with porewaters warmed at low tide during the day [25], or as a result of the interaction of overlying water flow and rippled beds or mounds [21]. Simultaneously, vertical flow can also be generated at ebb tide if the hydrostatic pressure is increased at depth and forces the porewaters to the surface [24]. Vertical flow is usually favored in marshes with flat topography, in coarse-grained sediments, in rippled sediments, or in confined zones such as creeks surrounded by banks. In turn, high lateral flow may occur if preexisting sediment structures, such as shallow confined permeable layers [22] or mounds [23], force a portion of the tidal flow in the horizontal direction.

The complexity of hydrological processes in intertidal salt marsh sediments coupled with the generally significant bioturbation, the high content of organic matter, and large sediment heterogeneities probably affect biogeochemical processes over a variety of spatial and temporal scales. As a result, new strategies to investigate these processes with a high spatial and temporal resolution have to be adopted. Voltammetric techniques are attractive to measure redox chemical species involved in diagenetic processes in situ because several analytes can be detected simultaneously, their detection limits are reasonably low, and high spatial and temporal resolutions can be achieved [26].

Brendel and Luther [27] developed a mercury-gold amalgam (Au/Hg) voltammetric microelectrode for the quantification of dissolved O_2(aq)_, Fe^2+^, Mn^2+^, and ΣH_2_S as well as the qualitative determination of FeS_(aq) _[28] and soluble organic-Fe(III) complexes [30] that may be pervasive in marine sediments. Depth profiling in sediments have mostly included single electrode measurements after collecting sediment cores [e.g., [12,27,29,31-33]]. In turn, several microelectrodes have been used simultaneously to determine the three-dimensional distribution of redox chemical species in sediment cores [13] and in situ in salt marsh sediments [34], continental margin sediments [35,36], and deep-sea hydrothermal vents [37]. These techniques have yet to be used to study the dynamic biogeochemical cycling of redox chemical species in intertidal salt marsh sediments.

The objectives of this project were to investigate the role of tidal forcing on the biogeochemistry of intertidal salt marsh sediments. To show that traditional sediment sampling and ex situ analysis with high spatial resolution can provide information relevant to seasonal variations in intertidal sediments, we present four years of ex situ porewater measurements with Au/Hg voltammetric microelectrodes at two different sites in the same salt marsh. To demonstrate that tides may have an important effect on the biogeochemical signatures of porewaters and the solid phase over short time and spatial scales, we compare traditional sampling techniques and ex situ voltammetric analyses over a tidal cycle with in situ depth profiling with Au/Hg voltammetric microelectrodes. Finally, to determine the effect of tidal forcing on the biogeochemistry of salt marsh sediments we correlate in situ measurements obtained with several microelectrodes positioned at different depths and porewater movements measured during several tidal cycles at the two different sites.

## Sampling Site

**Figure 1:**
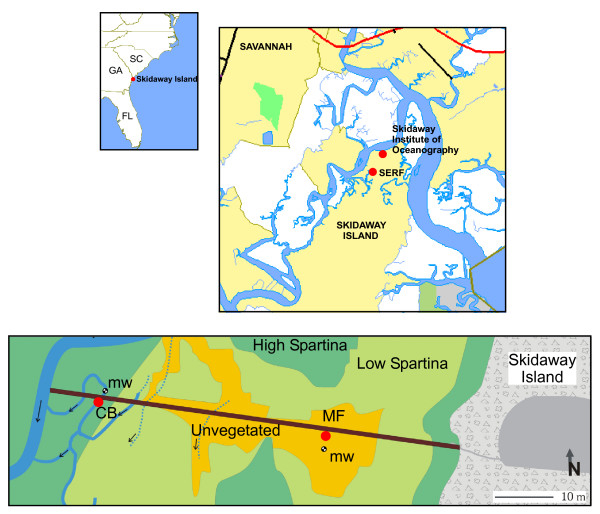
Geographic location of Skidaway Island, the Salt marsh Ecosystem Research Field station (SERF), and sampling sites along the boardwalk across the salt marsh. The mud flat site (MF) is located 122 m from the island in the unvegetated part of the salt marsh. The creek bank site (CB) is located 168 m from the island in a perennial channel off the main creek. Four monitoring wells (mw), positioned approximately 30 cm apart, were inserted at each site to monitor fluid advection. Arrows indicate direction of the surface water flow at ebb tide.

The study was conducted at the Salt marsh Ecosystem Research Facility (SERF) in the Skidaway Island salt marsh on Skidaway Island, Georgia (USA). Skidaway Island is located off the Georgia coast, approximately 5 miles south of Savannah (Figure 1). The salt marshes on the East Coast of North America cover an area of 589,429 ha [38] and developed in an intertidal region in the back of a barrier-island system. In general, the sedimentological composition of salt marshes in the Georgia bight are characterized by muddy sediments. These deposits are part of the Pleistocene/Holocene Satilla Formation [39] and of the Holocene Silver-Bluff terrace [40]. This terrace marks the last sea level highstand of about 1.8 m above present day sea level.

SERF includes a 213 m long boardwalk which provides direct access to the Skidaway Island salt marsh. This boardwalk spans a range of environments, from an inland salt marsh meadow to a tidal creek. This area is isolated from the nearby river (Figure 1) and not exposed to wave actions created by boats or winds. All the measurements reported were performed in the low marsh, where water levels vary between 1.5 m for neap tides and 2 m for spring tides (USGS web site). More than fifty small sediment cores were collected along the boardwalk and analyzed for porewater and solid phase composition over a period of four years at SERF (Table 1). This study reports analyses performed at two sites along the boardwalk only (Figure 1): The mud flat site (MF), located 122 m from the island; and the creek bank site (CB), located 168 m from the island. The MF site is centered in an approximately 400 m^2 ^unvegetated area of the marsh. In 2005, short *Spartina *began to grow in the area of study, but its density is not as high as other areas of the marsh. The sediment at MF is flat and exposed to the atmosphere for about 6 hours during each tidal cycle. Several small perennial creeks supply water to the site at rising tide. As a result, the water reaches the site as a front. The CB site is located in a perennial creek adjacent to the main creek at the end of the boardwalk (Figure 1). The creek is about 2.5 m wide at high tide and 1.5 m deep. Its banks are unvegetated but surrounded by a high *Spartina *field. The bottom of the creek is flat and about 1–2 m wide. Its sediment bed, where all the measurements were performed, is exposed to the atmosphere for about 3 hours per tidal cycle. The water is supplied to the site at rising tide through the main adjacent creek. Grain size analyses showed that 50% in weight of both sediments are well sorted fine grained sediments (< 75 μm in size). In average, the sizes of 10% of the sediment (d_10_) and 20% of the sediment (d_20_) are approximately 3 and 17 μm, which correspond to silty clays [41].

**Table 1:** Sites description, overlying water conditions, and properties of the sediment cores collected at SERF between 2001 and 2004.

			**Overlying Water**		**Sediment Core**		
**Site**	**Sampling Date**	**Tide State**	**Salinity**	**Temperature [°C]**	**Max. Depth [mm]**	**Total Fe [μ moles]**	**Total S [μ moles]**
MF	3/7/2001	Low	26	15	43	0	0.6
MF	3/7/2001	Low	26	15	48	0	4.16
MF	3/7/2001	Low	26	15	46	0	8.61
MF	3/7/2001	Low	26	15	48	0	18
MF	3/7/2001	Low	26	15	47	0	22.63
MF	6/11/2001	Low	30	30.2	33	43.00	46.04
CB	6/12/2001	Low	28	29.7	75	21.53	0
MF	7/13/2001	High	29	27.8	35	0	32.57
CB	7/14/2001	Low	28	28.5	40	14.71	0
CB	6/11/2002	Low	33	27.6	51	26.71	0.43
MF	6/12/2002	Ebb	31	27.7	63	0	122
CB	6/14/2002	High	30	29	72	106.53	0.15
CB	6/14/2002	Low	30	33.7	70.6	11.73	0.39
CB	6/14/2002	Rising	30	29.3	70	33.09	0.20
CB	6/16/2002	Low	32	27.5	85.4	56.43	0.04
CB	6/27/2003	High	19	25.9	70.5	8.79	0.11
CB	7/8/2004	Low	31	23.5	64	3.48	0.03
CB	7/9/2004	High	30	24	35	27.64	0

## Methods

At each of the MF and CB sites, four monitoring wells were installed in a row, 30 cm apart from each other, to monitor porewater pressures as a function of depth and time in the sediment during tidal cycles. Each well was made of 3 m long PVC pipe of 3.8 cm diameter sealed at the bottom and contained a single 0.15 mm slotted screen approximately 5 cm long positioned at different interval from the bottom of the wells (i.e., 120, 105, 90, and 60 cm). At each site, the four wells were gently inserted in the sediments such that the screens were located at the sediment-water interface (SWI), 15, 30, and 60 cm below the SWI. The wells were not submerged at high tide and were covered with lose fit caps to prevent potential interferences from precipitations and avoid overpressurization. Water levels were determined in the monitoring wells by pressure transducers (Leveloggers, Solinst) corrected for measured atmospheric pressure (Barologger, Solinst). Unless indicated, they were not corrected for the difference in screen height between the wells, such that differences of 15, 30, and 60 cm remain between the water levels of the well positioned at the SWI relative to the others at all time.

Ex situ geochemical measurements were obtained in sediment cores collected directly from the boardwalk with a sediment corer made of a 50 cm long and 7.5 cm diameter polyacrylic liner closed with a polyacrylic cap. The cap contained an exhaust that was opened and sealed remotely when the corer was, respectively, inserted in and removed from the sediment. A long pole was connected to the sediment corer to collect submerged sediment from the water surface. The sediment was removed by gently pulling the corer while maintaining the cap sealed. Coring at each site was performed within a 1 to 2 m radius when sampling periods were too close to each other to limit artifacts due to the removal of sediment. Sediment cores were carefully transported to the laboratory located less than a kilometer away. When possible, sediments were collected with a large volume of overlying water to avoid disturbing the SWI. At low tide, when the volume of overlying water was small, capping the sediment with a rubber plug helped in preserving the integrity of the SWI during the short trip to the laboratory.

Ex situ and in situ voltammetric measurements were obtained with mercury-plated gold (Hg/Au) microelectrodes. A three electrode system, consisting of an Ag/AgCl reference electrode, the Hg/Au microelectrode as working electrode, and a Pt counter electrode, was used for these measurements. Ex situ depth profile measurements were performed within 30 minutes after collection with a computer-operated DLK-100 potentiostat and a portable micromanipulator that can be piloted remotely by the computer (Analytical Instrument Systems, Inc.). For these measurements, a working electrode was positioned in an electrode holder and the reference and counter electrodes were attached to the side of the core liner such that they remained in the overlying waters [13]. The electrode was positioned above the SWI, and voltammograms were recorded as a function of depth in at least triplicates. In situ measurements were conducted with a 50 by 100 cm rectangle benthic lander specifically designed for deployments from the board walk. The four-footed lander was fitted with a voltammetric system to analyze the porewater composition as a function of depth and time over tidal cycles. The In Situ Electrochemical Analyzer (ISEA, Analytical Instrument Systems, Inc.) consisted of a potentiostat encased in a waterproof pressure housing and an underwater micromanipulator (Analytical Instrument Systems, Inc.) operated by the potentiostat. With this system, up to eight voltammetric electrodes can be deployed in situ with a holder that carries all the electrodes within a 3 cm diameter circle. In situ depth profiles were first collected with a single working electrode that was lowered with vertical increments varying between 1 and 2 mm. For these measurements the reference and counter electrodes were positioned in a separate holder against one of the feet of the lander such that they were always located in the overlying water. The position of the SWI was estimated from the sharp decrease observed in the voltammetric noise upon reaching the sediment surface. In a second series of in situ measurements, an array of up to five electrodes were deployed at fixed depths in the sediment to collect time series data during tidal cycles. For each of the time series deployments, the tip of the uppermost working electrode was positioned at a known height from the tips of the reference and counter electrodes on the same electrode holder, and the tips of the remaining electrodes were positioned at fixed heights from each other. The micromanipulator was then lowered to position the reference and counter electrodes at the SWI. The lander was always deployed at low tide, when the sediment was exposed to the atmosphere, such that the reference and counter electrodes could be positioned as close as possible to the SWI using a coarse micromanipulator displacement setting. The electrode system was then lowered with a fine millimeter resolution, and voltammetric scans were acquired after each spatial increment until the electrodes made contact with the SWI. The SWI was inferred to be the location at low tide where the counter and reference electrodes were in electrical contact with the uppermost working electrode.

Au/Hg working microelectrodes were manufactured as previously described [30-32] and calibrated by the pilot ion method [27]. Working microelectrodes were fabricated from 4 mm diameter hollow glass pulled at one end under high temperature to form a tip of about 5 cm length and 1 mm diameter. A 100 μm gold wire was sealed into the glass tip with an Epoxy resin (West Marine) and contacted to a copper wire with Ag solder. Using this technique, the electrodes were rigid and not influenced by water flow during tidal cycles. The electrode tip was sanded to a disk and then polished successively with 15, 6, 1, and 0.25 μm diamond pastes (Buehler). The gold disk was plated in a 0.1 M Hg(NO_3_)_2 _solution for 4 minutes. The mercury film was then conditioned at -9 V for 90 seconds to form a better amalgam between the gold and the mercury. Linear sweep voltammetry (LSV) was exclusively used to measure dissolved oxygen (MDL ~4 μM), while cathodic square wave voltammetry (CSWV) was used to quantify Mn^2+ ^(MDL ~15 μM), Fe^2+ ^(MDL ~25 μM), and ΣH_2_S (= H_2_S + HS^- ^+ S^2- ^+ S_x_^2- ^+ S(0), MDL ~0.2 μM) and detect FeS_(aq) _[28] and soluble organic complexes of Fe(III) [30]. For these measurements, the potential was scanned from -0.1 V to -1.8 V after a conditioning period of 10 s at -0.1 V to clean the electrode between measurements. When soluble organic Fe(III) complexes or dissolved sulfide were present, a conditioning step was also applied at -0.9 V for 10 s to remove these species from the electrode surface before the next measurement. When the concentration of dissolved sulfide was high, anodic square wave voltammetry (ASWV) was preferred to avoid formation of HgS double films [42]. Scan rates of 200 mV/s were typically applied for all measurements. The chemical composition of soluble organic-Fe(III) complexes and FeS_(aq) _are still unknown and cannot be quantified using external calibrations. These species are therefore reported in voltammetric current intensities. Voltammetric data were processed with a home-built Matlab program [43] that integrates peaks (SWV) and derives waves (LSV). When voltammetric signals for Mn^2+ ^and Fe^2+ ^overlapped, their currents were deconvoluted assuming voltammetric peaks display gaussian shapes [43]. Average and standard deviations reported represent a minimum of triplicate measurements.

After in situ voltammetric measurements, a sediment core was retrieved and sectioned in depth increments of about 5 mm to determine the chemical composition of the solid phase and measure porewater species not detectable by voltammetry (i.e. orthophosphate, nitrate, nitrite, total dissolved inorganic carbon). When ex situ voltammetric measurements were conducted, the same core was sectioned for porewater analyses, generally within a couple of hours after collection (the time it takes to complete ex-situ voltammetric measurements). All sediment processing was performed in a polypropylene glove bag (Aldrich) under N_2 _atmosphere to avoid oxidation of reduced chemical species. Once sectioned, sediment layers were placed in 50 ml centrifuge tubes (Nalgene) and centrifuged at 3000 rpm under N_2 _atmosphere for 5 to 10 minutes to separate the porewaters from the solid phase. Porewaters were collected with acid-washed polypropylene syringes (Norm-Ject, Henke Sass Wolf) and immediately filtered in the glove bag through 0.2 μm polysulfone membrane Puradisc filters (Whatman). The filtrate was then split for immediate analysis (i.e., total dissolved inorganic carbon, orthophosphate) or frozen for further analysis, generally within 48 hours (i.e., nitrate, nitrite). The remaining sediment was sealed under N_2 _and frozen until analysis.

Orthophosphates (ΣPO_4_^3-^) were measured in the porewaters by spectrophotometry [44]. Amorphous iron oxides and total reactive iron were determined in the solid sediment using the ascorbate and dithionite methods [45]. The ascorbate reagent extracts only amorphous forms of Fe^3+ ^whereas the dithionite extractant dissolves amorphous iron oxides, acid volatile sulfide (AVS), and crystalline iron oxides including magnetite, goethite, and to a lesser extent chlorite [45]. Triplicate wet sediment samples were leached with ascorbate at 25°C for approximately twenty four hours and with dithionite for approximately four hours in a 60°C water bath. Samples were then filtered, diluted, and analyzed by the ferrozine method [46]. Acid volatile sulfide (AVS) was extracted in triplicate samples by cold distillation in 3 mol L^-1 ^HCl under a N_2 _atmosphere [47]. The volatile H_2_S gas was distilled for four hours and trapped in a 1 mol L^-1 ^NaOH solution. An aliquot of this solution was then analyzed voltammetrically for ΣH_2_S in 0.5 mol L^-1 ^NaCl.

Wet sediment cores from both sites were sectioned in layers of 5 to 10 mm and sieved sequentially in three different size fractions: >125 μm, 63–125 μm, and <63 μm. The smallest fraction of each layer was dried and analyzed using a particle analyzer (SediGraph 5100 V2.02). The particle size distribution in these fractions was used to estimate the hydraulic conductivity (K) of each sediment layer based on the empirical relationship for well-sorted sediments [48]:

K = 0.36(d_20_)^2.3^

where d_20 _represents the average diameter of 20% of the particles in these sediments in mm, and K is the hydraulic conductivity in cm s^-1^.

## Results

**Figure 2:**
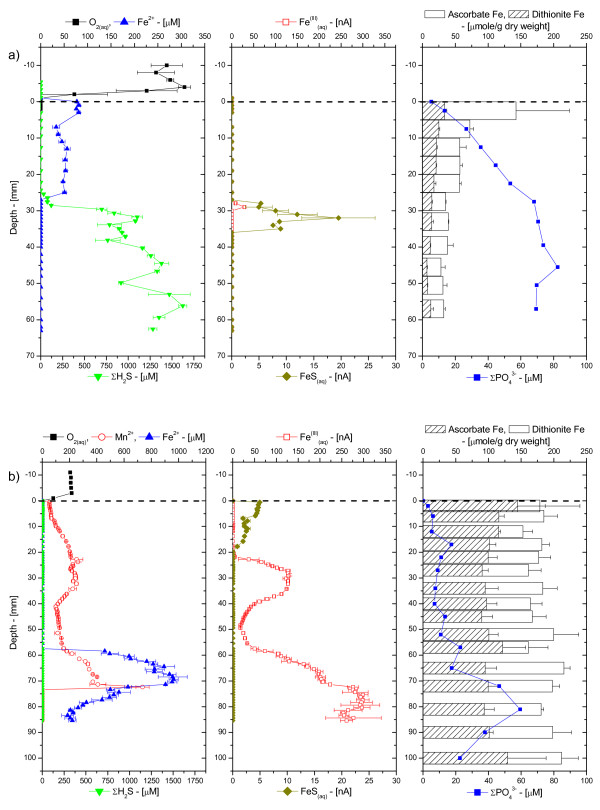
Porewater O_2(aq)_, Mn^2+^, Fe^2+^, ΣH_2_S, org-Fe^(III)^_(aq)_, FeS_(aq)_, and ΣPO_4_^3-^, and ascorbate- and dithionite-extractable Fe as a function of depth in sediment cores collected ex situ at: a) the mud flat site; and b) the creek bank site in the Skidaway Salt Marsh in June 2002. All porewater chemical species, except ΣPO_4_^3-^, were measured electrochemically in intact sediment cores. Mn^2+ ^was below minimum detection limit (MDL) in the mud flat porewaters. Note the difference in the O_2(aq) _and Fe^2+ ^concentration scales between both sites.

Generally, porewater profiles displayed contrasting chemistries over the first 10 cm of sediment at the MF and CB sites, as shown in the typical examples of Figure 2. In MF sediments, porewater profiles generally contained lower concentrations of Mn^2+ ^and Fe^2+^, but larger concentrations of dissolved sulfide (Figure 2a). At the CB site, porewaters contained low concentrations of dissolved sulfide, but larger concentrations of Mn^2+ ^and Fe^2+ ^(Figure 2a). In addition, soluble organic-Fe(III) complexes were only detected in CB sediments, while FeS_(aq) _was generally observed below the onset of dissolved sulfide. Sometimes, FeS_(aq) _was detected close to the SWI, even in CB sediments (e.g., Figure 2b). Total dissolved orthophosphate, while generally around the same concentration, also exhibited characteristic profiles at both sites. In MF sediments, total dissolved orthophosphate reached the SWI, while in CB sediments, it was mostly present in the deep porewaters (Figure 2). The solid phase also displayed contrasting depth profiles at both sites. In MF sediments, the concentrations of total extractable iron (dithionite Fe) and amorphous iron oxides (ascorbate Fe) were low with maxima at the SWI (Figure 2a). In CB sediments, the concentrations of ascorbate and dithionite Fe were both high and homogeneously distributed (Figure 2b).

**Figure 3:**
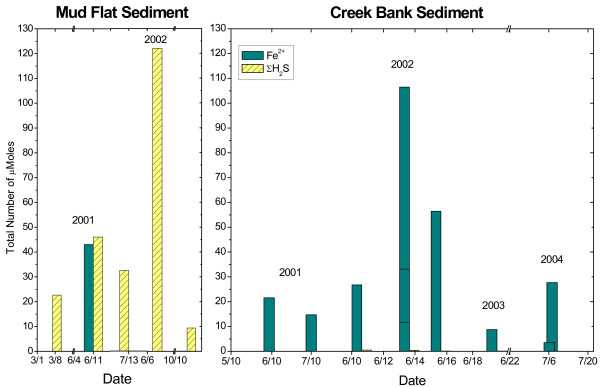
Depth-integrated Fe^2+ ^and ΣH_2_S mole content of the porewaters at the creek bank and mud flat sites over the last four years. The number of moles was calculated assuming the porewater homogenous over the volume defined by the sediment core diameter and the vertical distance between measurements.

To compare geochemistries at both locations, depth-integrated mole contents of Fe^2+ ^and ΣH_2_S were determined in each sediment core by summing, over the entire length of the sediment core, the number of moles measured in the volume of sediment delimited by the depth interval between measurements and the surface area of the sediment core. These calculations assume that the concentrations measured electrochemically represent the average concentrations in each slice and that the porosity changes as a function of depth do not affect the concentrations significantly. The depth-integrated mole content of Fe^2+ ^and ΣH_2_S in the cores collected seasonally between 2001 and 2004 were clearly distinct at both sites (Table 1 and Figure 3). MF sediments contained high dissolved sulfide and low ferrous iron concentrations, while CB sediments were characterized by high concentrations of reduced iron and low concentrations of dissolved sulfide.

**Figure 4:**
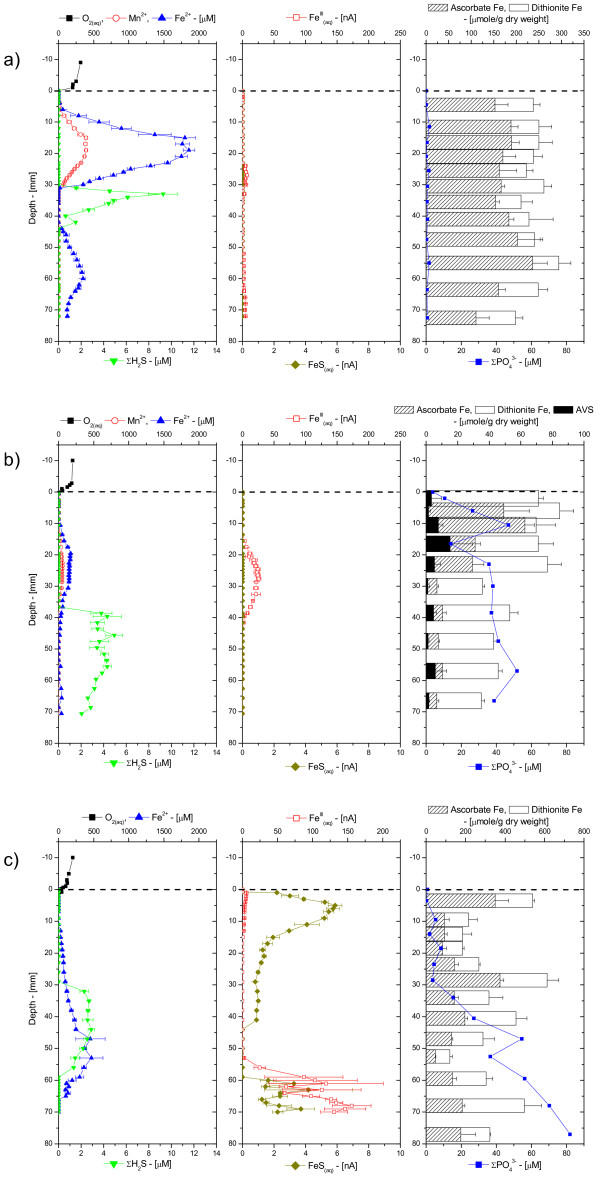
Porewater O_2(aq)_, Mn^2+^, Fe^2+^, ΣH_2_S, org-Fe^(III)^_(aq)_, FeS_(aq)_, and ΣPO_4_^3-^, and ascorbate- and dithionite-extractable Fe as a function of depth in sediment cores collected ex situ at the creek bank site in the Skidaway Salt marsh at: a) High tide; b) Low tide; and c) Rising tide over a 24 hour period in June 2002. Acid volatile sulfide (AVS) was quantified in the low tide sediment core only.

In an attempt to determine the role of tidal forcing on the biogeochemical processes in porewaters, a sediment core was collected from the CB site at three different times during the same tidal cycle (Figure 4). The first core, collected at high tide, displayed extremely high concentrations of both Mn^2+ ^and Fe^2+ ^right below the SWI and a dissolved sulfide peak between 30 and 40 mm that reached relatively low concentrations (i.e. < 10 μM). Below 40 mm, Fe^2+ ^was produced again, while Mn^2+ ^had totally disappeared from the porewaters (Figure 4a). This core also generated low voltammetric currents for soluble organic-Fe(III) and FeS_(aq)_. The second core, collected at low tide, displayed a similar profile but with much lower concentrations in all the dissolved species mentioned above, except for the presence of a stronger voltammetric signal for soluble organic-Fe(III) between 20 and 40 mm (Figure 4b). At the next flooding tide, porewater profiles looked completely different, with dissolved Fe^2+ ^that progressively increased with depth and reached a maximum concentration of 500 μM at 55 mm. Interestingly, the onset of dissolved sulfide was found at approximately the same location and range of concentrations as the other two cores (Figure 4c), while aqueous FeS showed a maximum just below the sediment water interface, was depleted at the depth of maximum dissolved sulfide, and increased below the zone of dissolved sulfide minimum. Soluble organic-Fe(III) was small near the SWI and increased below the sulfide minimum (Figure 4c). Total dissolved orthophosphate varied significantly during the tidal cycle. At high tide, it was close to detection limit (Figure 4a), at low tide it reached the SWI and displayed a typical transient diffusive profile (Figure 4b), while at the following flooding tide, it exhibited the same transient diffusive profile but with an onset at 30 mm below the SWI (Figure 4c). Finally, the solid phase of the same sediment cores revealed curious heterogeneities in the creek. While its total reactive and amorphous iron oxides content were high and relatively constant with depth at high and flooding tide (Figure 4a and 4c), they were much lower in concentration at low tide and displayed a maximum in the first 20 mm of sediment (Figure 4b). AVS measurements performed at low tide revealed low concentrations of FeS_(s) _in these surficial sediments. Since these sediment cores were collected within one meter of each other, these differences can only be attributed to lateral heterogeneities within the creek. The measurements clearly showed that the influence of tidal forcing on the biogeochemical processes in porewaters could not be considered using conventional sampling and analyses because sediment heterogeneities could not be discerned from temporal variations during tidal cycles.

**Figure 5:**
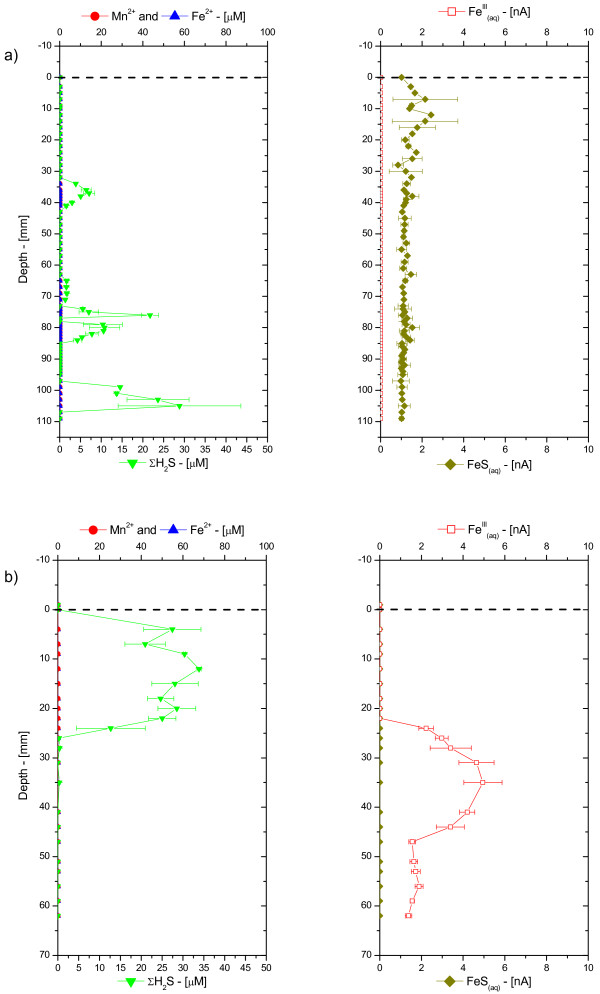
Porewater Fe^2+^, ΣH_2_S, org-Fe^(III)^_(aq)_, and FeS_(aq) _measured in situ as a function of depth with voltammetric Au/Hg microelectrodes at: a) the mud flat site starting at low tide; and b) the creek bank site starting at high tide in the Skidaway salt marsh in October 2002. Mn^2+ ^was not detected in both sediments.

In situ depth-profile measurements were conducted at both sites to capture the effect of tidal forcing on the geochemistry of porewaters. Each profile was completed within four hours after deployment during the fall season, when sulfate reduction is usually in decline. Surprisingly, the MF and CB sites revealed relatively similar geochemistries (Figure 5), with no significant soluble organic-Fe(III) (< 5 nA), Mn^2+^, and Fe^2+ ^across the entire profiles, presence of relatively low concentrations of dissolved sulfides for salt marsh sediments (< 30 μM), and small but detectable levels of aqueous FeS. The accumulation of dissolved sulfide in the porewaters at both sites, on the other hand, displayed a rather distinct feature. In the MF sediment, it occurred sporadically at three distinct locations, 30, 75, and 105 mm below the SWI. In contrast in the CB sediment, it formed a peak 15 mm below the SWI, just above the soluble organic-Fe(III) maximum.

**Figure 6:**
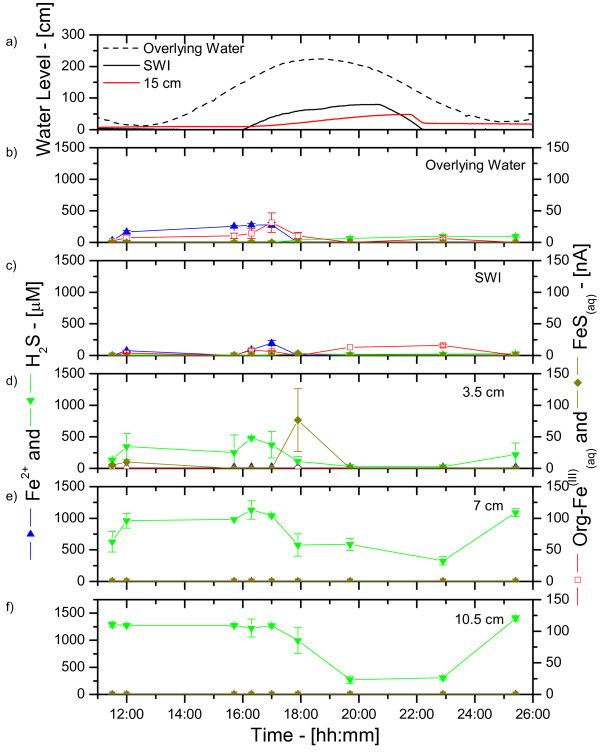
Water level changes (from low tide levels) and porewater Mn^2+^, Fe^2+^, ΣH_2_S, org-Fe^(III)^_(aq)_, and FeS_(aq) _measured in situ at five fixed depths and as a function of time during a tidal cycle at the mud flat site in June 2003. a) Well water levels measured at the sediment-water interface (SWI) and 15 cm below the SWI, and tidal fluctuations obtained from the Fort Pulaski NOAA buoy; Concentrations (left) and current intensities (right) measured: b) in the overlying water; c) at the SWI; d) at 3.5 cm; e) at 7.0 cm; and f) at 10.5 cm. Dissolved oxygen was never detected in the porewaters.

**Figure 7:**
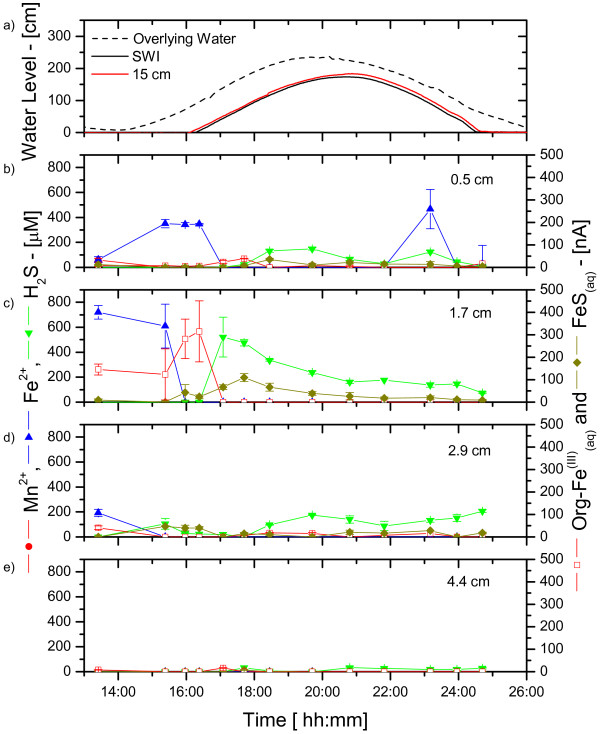
Water levels changes (from low tide levels) and porewater Mn^2+^, Fe^2+^, ΣH_2_S, org-Fe^(III)^_(aq)_, and FeS_(aq) _measured in situ at four fixed depths and as a function of time during a tidal cycle at the creek bank site in June 2003. a) Well water level changes (from low tide levels) measured at the sediment-water interface (SWI) and 15 cm below the SWI, and tidal fluctuations obtained from the Fort Pulaski NOAA buoy; Porewater concentration (left) and current intensities (right) at: b) 0.5 cm; c) 1.7 cm; d) 2.9 cm; and e) 4.4 cm. Dissolved oxygen was never detected in the porewaters.

Finally, several series of in situ measurements were obtained during tidal cycles using up to five electrodes positioned at different depths in the MF and CB sediments. Figure 6 and Figure 7 display two examples of such measurements obtained within days of each other. These time series illustrated the extreme complexity of intertidal salt marsh sediments. They revealed rapid changes in concentrations of Fe^2+^, dissolved sulfide, and current intensities of soluble organic-Fe(III) and FeS at each depth that seemed to correlate with water levels measured in the sediment with the monitoring wells and water levels above the sediment obtained from the NOAA buoy at Fort Pulaski. At the same time, they showed strong variations with depth, probably related to the heterogeneity of sediments and the complex hydrologies at these sites. Nonetheless, important trends could be observed during tidal cycles. At low tide (before 15:30 and after 22:00), the MF sediment displayed high concentrations of reduced species (i.e., Fe^2+ ^and ΣH_2_S) at most of the depths surveyed (Figure 6). At rising tide, ΣH_2_S seemed to initially be produced at depth (Figure 6d–f), then consistently removed, while simultaneously produced at low concentrations in the overlying waters. Similarly, Fe^2+ ^was initially produced at the SWI and the overlying waters at rising tide (Figure 6b–c) but was removed completely when ΣH_2_S reached the overlying waters. Except at 3.5 cm, the removal of sulfide was not accompanied by the production of FeS_(aq) _complexes at depth. As these complexes are precursors in the precipitation of FeS_(s) _[28], precipitation of iron sulfide minerals did not appear responsible for the decrease in ΣH_2_S and Fe^2+ ^concentrations at rising tide, at least at the time of these measurements. These data instead suggest that porewater advection at rising tide pushed reduced species towards the SWI where Fe^2+^, and possibly ΣH_2_S, were oxidized by dissolved oxygen. The oxidation of Fe^2+ ^was supported by the production of soluble organic-Fe(III) complexes at the SWI and in the overlying waters at rising tide (Figure 6b–c). At ebb tide, the concentrations of these species did either not change or decreased slightly as a function of time (Figure 6).

CB sediments behaved in a similar fashion. The concentration of reduced chemical species was generally high just below the SWI at low tide (Figure 7b–c) but changed at rising tide. First, Fe^2+ ^was abruptly removed from the surficial porewaters at the onset of the tide change, before ΣH_2_S appeared in the sediment layers. Interestingly, the removal of Fe^2+ ^occurred at 1.7 cm first, then at 0.5 cm, and was accompanied by the production of soluble organic-Fe(III) (Figure 7b–c), suggesting again that oxidation of Fe^2+ ^took place in surficial sediments. Dissolved sulfide was initially produced at depth at rising tide (Figure 7b–d), then was slowly removed from the porewaters. The removal of ΣH_2_S was accompanied by the production of FeS_(aq) _complexes close to the SWI (Figure 7b–c), suggesting that precipitation of FeS_(s) _could occur in CB sediments. At ebb tide, the concentrations of Fe^2+ ^and ΣH_2_S as well as current intensities of FeS_(aq) _and soluble organic-Fe(III) complexes did not change significantly (Figure 7b–e).

**Figure 8:**
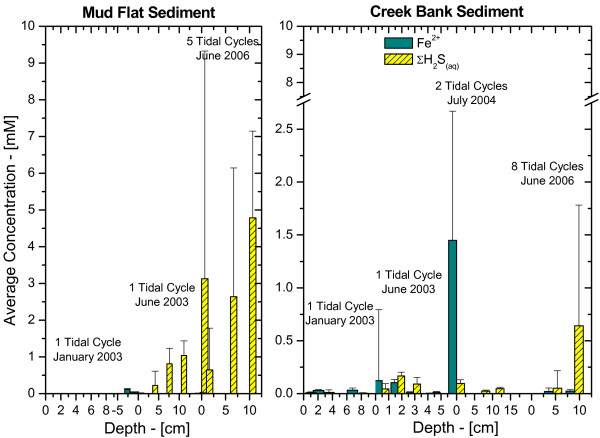
Average concentrations of Fe^2+ ^and ΣH_2_S measured in situ during tidal cycles as a function of depth at: a) the mud flat site; and b) the creek bank site. The standard deviations represent temporal variations from the average concentration during tidal cycles. The number of tidal cycles during which concentrations were measured and the date of these measurements are provided above each data set.

Similar in situ measurements were conducted over three years and at different seasons at the same sites. To compare the behavior of the different sites over tidal cycles, the average Fe^2+ ^and ΣH_2_S concentrations measured as a function of time at each depth and both sites was represented with the temporal deviation from their average concentrations as a function of depth in the sediment (Figure 8). This comparison confirmed the high temporal variations linked to tidal changes and revealed two main differences between MF and CB sediments. First, MF sediments displayed higher concentrations of dissolved sulfide compared to the CB site, which could produce relatively high concentrations of Fe^2+ ^(Figure 8). Second, the concentration of dissolved sulfides increased regularly with depth in MF sediments, while concentrations were much more variable with depth in CB sediments.

**Figure 9:**
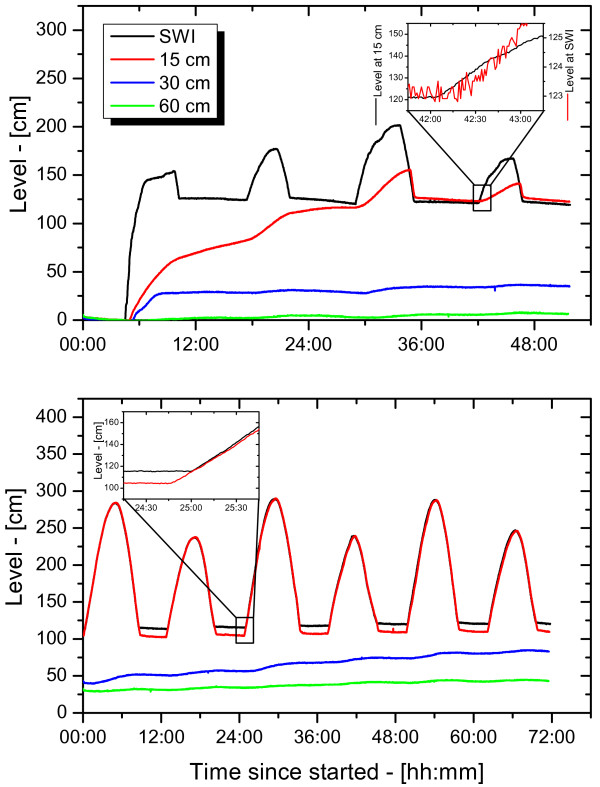
Water levels in the monitoring wells at the sediment-water interface (SWI), 15, 30, and 60 cm below the SWI recorded as a function of time in the: a) mud flat and b) creek bank sites in June 2003. Measurements were collected immediately after the wells were installed at the mud flat site, while measurements were collected two days after installation at the creek bank site. Insets show a close view of the differences in water levels at both sites.

To correlate the geochemical changes with the movement of porewaters during tidal cycles, water levels were recorded in the monitoring wells adjacent to each site over more than three years, from June 2003 to August 2006. The change in water levels in the wells during tidal cycles was due to the variation in hydrostatic pressure in the sediment over time and reflected the movement of water in the sediment pores. MF water levels, monitored immediately after the wells were installed, indicated that the SWI and 15 cm wells reached equilibration after approximately 30 hours (Figure 9a). CB water levels were monitored only 52 hours after their installation and indicated that the SWI and 15 cm wells were already at equilibrium (Figure 9b). In turn, water levels deeper than 30 cm in both the CB and MF sediments did not change significantly over tidal cycles, even after several months of equilibration (not shown), suggesting that the permeability of these sediments was too low below that depth to be susceptible to tidal variations. Interestingly, water levels obtained at 15 cm deep and near the SWI indicated that the hydrology at both sites was different. While the water levels in both wells at the CB site varied at relatively the same rate (Figure 9b), water levels at the SWI changed more rapidly than 15 cm below the SWI in MF sediments (Figure 9a). In addition, the relative change in water levels between low and high tide were smaller in MF than CB sediments. Finally, an approximate 15 minute phase lag was observed between the time the water rose in the SWI and 15 cm wells at the MF site (inset in Figure 9a); in contrast, a 15 minute lag was found between the 15 cm and SWI wells at the CB site (inset in Figure 9b). These data indicate that water percolated from the sediment surface to deeper depths at the MF site, while water advected from depth to the surface of the sediment at the CB site.

## Discussion

Intertidal salt marsh sediments are extraordinarily complex environments that display extremely high temporal and spatial variabilities. Unfortunately, techniques able to measure multiple redox species simultaneously with a high temporal resolution and in several dimensions do not presently exist. The results of the present study were obtained with a combination of voltammetric microelectrode and conventional porewater and solid phase analyses to gain better insights into the role of tidal forcing on the biogeochemistry of these sedimentary environments near the sediment-water interface. The measurements, together with the numerous data obtained over the last four years at SERF [13,41,49-51] show that, in general, mud flat sediments are more reducing environments, with larger sulfide concentrations produced than in creek bank sediments, at least during the warm season (Table 1, Figure 3). In contrast, creek bank sediments contain large concentrations of ferrous iron (Table 1, Figure 3) and sometimes reduced manganese (Figure 2, Figure 4).

Measurements of water levels (e.g., Figure 9) and temperature (not shown) in the monitoring wells of the MF and CB sites over more than three years indicate that the porewaters deeper than 30 cm below the SWI at both sites are almost not influenced by tidal fluctuations. These data suggest that the permeability of these sediments generally decreases below that depth and limits water exchange during tidal cycles (Figure 9). Porosities measured in unvegetated sediment cores confirm these findings: They vary between 86(+/-4)% at the surface to 70(+/-5)% around 50 cm and display a significant decrease below 20–30 cm depending on the sites (Clark Alexander, personal communication). As most geochemical investigations use sediment cores of less than 50 cm, conventional ex situ measurements in intertidal sediments should be carefully considered if the sediments are collected during flood or ebb tide and probably accompanied by a hydrological survey. In contrast, well water measurements over the first 30 centimeters of sediment reveal significantly different hydrologies at the MF and CB sites. First, the MF wells equilibrate at a slower rate than the CB wells (e.g., Figure 9), suggesting that hydrostatic pressure gradients in mud flat sediments are lower than in creek sediments. Second, the average exposure time of the sediment to the atmosphere at low tide, obtained from five series of measurements over more than three years, is about twice as much longer at the MF site (366(+/-64) min) than the CB site (184(+/-74) min). Finally, the delay between the time at which water levels change in the SWI and 15 cm wells during rising tide at both sites indicates that porewaters advect in the vertical direction at both sites, though water seems to percolate from the surface in the mud flat while porewater is transported from the deeper depths to the sediment surface in creek sediments. Because of the close proximity of the four different wells at each site (less than 15 cm apart), water levels only provide the vertical component of the fluid advection, and we cannot exclude the fact that porewater may overall move in the horizontal direction with a significant vertical contribution. Nevertheless, the difference in hydrostatic pressure gradients at the CB and MF sites can be explained if hydraulic conductivities are distinct. Interestingly, hydraulic conductivities estimated from the particle sizes and Eq. 1 vary approximately between 6 10^-7 ^and 6 10^-5 ^cm s^-1 ^with depth in the sediment at both sites (not shown). Given the level of incertitude of these measurements, these variations are not significant between sites. They compare well with conductivities of other fine-grained salt marsh sediments [52], but are much lower than sandy salt marsh sediments with high groundwater inputs [22]. Alternately, the difference in hydrologic pressures at the CB and MF sites could be explained if the topography of the marsh affects physical processes [17]. The CB site is closer to the estuary (Figure 1) and at a slightly lower elevation than the MF site. Channels in salt marshes form during the tidally-related meandering of water. High energy waters erode undercut slopes and facilitate deposition of fine-grained sediments at slip-off slopes [53]. These high energies bring coarse-grained sediments that are preferentially deposited at the bottom of channels. This process repeated over tidal cycles slowly generates creeks with high-permeability bottoms that are surrounded by banks with progressively lower permeabilities [52]. Over time, fine-grained sediments accumulate on creek banks and creates areas of high elevation, where *Spartina *can develop. The presence of *Spartina *in the high marsh near the adjacent creeks must decrease the overall permeability of the sediments in which it grows and thus limit the transport of porewaters through the vegetated creek bank sediment [20]. As a result, the flow field from the nearby river at rising tide (Figure 1) must increase the hydrostatic pressure in the neighboring subsurface which, in turn, should promote a three dimensional displacement of the porewaters away from the incoming tide. If the surface area of the unvegetated creek sediment is smaller than that of the main tidal river, the hydrostatic pressure should be higher in the unvegetated creek bank sediment, and a vertical displacement toward the sediment-water interface may be promoted. In contrast, the greater surface area above ground in mud flats may dissipate the energy flow at rising tide and decrease the hydrostatic pressure in the subsurface. In these areas, the overlying water is not confined by banks and floats over the sediment. Once the hydrostatic pressure is high enough above the sediment, overlying waters slowly percolate and displace porewaters deep in the sediment as shown by the longer time it takes to move water in the mud flat sediment (Figure 9).

At low tide, porewaters are static and biogeochemical reactions should result in the local accumulation of reduced chemical species. Ex situ depth-profile measurements (Figure 2 and Figure 4), even performed within 30 minutes after sampling, display significantly more complex geochemistries than our in situ profiles and time series, suggesting that sediment cores may be impacted very rapidly when the advective flux of porewaters is suppressed after its collection. At flood or ebb tide, the rate of biogeochemical reactions should not change locally, but the movement of porewaters may transport reduced chemical species, potentially diluting or concentrating the porewaters. In this scenario, reduced chemical species are likely to build up twice as much in mud flat sediments compared to creek sediments during each tidal cycle. Our in situ profiling measurements corroborate this theory. They show that dissolved sulfide can occasionally reach the SWI at rising tide, even in creek sediments (Figure 5b). Yet in situ time series data collected during up to four times over three years at both sites show that generally mud flat sediments produce much more dissolved sulfides and much less ferrous iron than creek sediments (Figure 8). Interestingly, the temporal variations of both species during tidal cycles are high at both sites, indicating that tidal forcing influences the first few centimeters below the sediment-water interface significantly. For example, the production of high concentrations of dissolved sulfide at depth at low tide (Figure 6e–f), followed by its removal when the hydrostatic pressure increases in the porewaters (both during flood and ebb tide) and reappearance at the next low tide indicate that sulfate reduction is active in the deep sediment layers and that tidal variations influence the position of the sulfide gradient in the sediments. However, these variations are not always related to changes in the direction of advection when the tides are reversed. In fact, data indicate that biological and geochemical reactions may largely influence the distribution of redox chemical species during tidal cycles. For example, the sudden rise and decrease in Fe^2+ ^concentrations at rising tide above 17 mm in CB sediments (Figure 7b and 7c) suggest that iron reduction provides episodic source of iron at depth or that its constant supply is episodically balanced by its removal. Ferrous iron could be removed from the porewaters when dissolved sulfide, produced during sulfate reduction in the deep sediment, advects and titrates Fe^2+ ^as FeS species (Figure 7b and 7c). Alternatively, dissolved sulfide could episodically advect to the surface of the sediment and reduce very reactive iron oxides while advecting. This scenario would account for the episodic production of Fe^2+^, as well as the absence of dissolved sulfide at rising tide in the intermediates depths (e.g., between 16:00 and 18:00 in Figure 7e) and its presence at the sediment surface at high tide (Figure 5b and Figure 7a).

These processes still do not explain the differences in redox state at the MF and CB sites, and in particular, how iron oxides are generated in creek sediments. Higher hydrostatic pressure gradients and a longer exposure to water movements during each tidal cycle in creeks could enhance oxygen penetration and change the redox state of creek sediments. The decrease in dissolved sulfide followed by the production of soluble organic-Fe(III) in the surficial sediment layers (≤ 3 cm) of the creek sediment at ebb tide (Figure 4a and 4b) and at rising tide (Figure 7) may be related to the input of dissolved oxygen when the hydrostatic pressure changes in the sediment. Interestingly, dissolved oxygen (i.e., > 5 μM) was never detected during tidal cycles, suggesting that if it penetrates the sediment, dissolved oxygen is readily removed by reduced chemical species. This explanation is corroborated by the quasi-absence of dissolved sulfide (< 25 μM), FeS_(aq) _(< 7 nA), as well as Mn^2+ ^(< 15 μM), and Fe^2+ ^(< 20 μM) at all depths in the sediment during ebb tide (Figure 5a) and the abrupt removal of Fe^2+ ^at rising tide (Figure 7b–c). Transfer of dissolved oxygen from the overlying water into the first 10 cm of sediment could be facilitated by bioturbation [11] and macrophytes [12]. These banks are surrounded by *S. alterniflora *that aerate surficial sediments through their roots. This supply of oxygen to the roots generally results in the precipitation and formation of large concretions of iron oxides [12]. Simultaneously, intertidal salt marsh sediments are highly bioturbated, especially by fiddler crabs and polychaetes [14,15]. Bioturbation actively brings dissolved oxygen to the first five centimeters of sediments in a random but highly dynamic fashion [11]. Permanent burrows also facilitate ventilation of the upper 10 cm of sediment either through wave action or, in our case, tidal forcing. This process should allow dissolved oxygen to penetrate the deep layers of sediments at ebb tide. Our measurements, however, have always been performed in unvegetated sediments, and infaunal bioturbation, at least to a first approximation, is not significantly different at the CB and MF sites. These measurements, therefore, suggest that the larger hydrostatic pressure gradients detected in creek compared to mud flat sediments are mainly responsible for the geochemical differences observed at both sites (Figure 8). This scenario could also explain why in situ profiling measurements obtained during ebb tide in CB sediments (Figure 5a) display high dissolved sulfide concentrations near the sediment surface but not deeper in the sediment, while the deep porewaters are, in contrast, dominated by large soluble organic-Fe(III) signals. Unfortunately, in situ profiling is relatively time-consuming and while the top 25 mm of sediment were analyzed near high tide, the deeper layers were probed during ebb tide.

In our high-spatial resolution study in salt marsh sediments [13], we found that Fe(III) reduction leads to the accumulation of Fe^2+ ^in the surficial porewaters (0–6 cm below the SWI) of CB sediments throughout the year, while dissolved sulfide was below detection limit. Even if sulfate and iron reduction were concomitant, the removal of porewater sulfide by oxidation or precipitation of FeS was most likely exceeded by chemical and/or microbial Fe(III) reduction. Because FeS precipitation rates are so fast, i.e. the half-life of H_2_S in the presence of Fe^2+ ^is in the order of milliseconds [54] versus hours to days in the presence of oxygen [e.g., [55]] or Fe(III) oxides [e.g., [56]], and because these sediments were neither black nor oxygenated at the time of our measurements, we concluded that sulfate reduction was not a significant terminal electron-accepting process in the top centimeters of these creek bank sediments. Thus, even if sulfate reduction is globally an important process linked to organic carbon oxidation, microbial Fe(III) reduction could certainly contribute to the cycling of carbon in these environments.

Indeed, several recent studies have suggested that microbial Fe(III) reduction may also contribute to carbon oxidation in salt marsh sediments, and Fe(III)-reducing bacteria have been detected in abundance in these environments [9,15,57-59]. The population density of Fe(III)-reducing bacteria was found to oscillate seasonally [16,59,60]: In summer, when sulfate reduction rates are high, the density of Fe(III)-reducing bacteria are low; in winter, when sulfate reduction rates are low, the density of Fe(III)-reducing bacteria rebound to high levels. Sulfate reduction rates and microbial iron reduction rates determined seasonally in vegetated and unvegetated salt marsh sediments [9,14,15] as well as mesocosms [14] have shown that microbial Fe(III) reduction may account for a significant fraction of carbon oxidation in surficial sediments that are heavily bioturbated. These findings suggest that anaerobic respiration of manganese and iron may depend on the recycling of manganese and iron oxides which is probably linked to physical processes.

Unless the anaerobic oxidation of Fe(II) [e.g., [61]] occurs at high rates in salt marsh sediments, the most plausible explanation for the recycling of iron oxides in the deep sediment layers (e.g., Figure 2b and Figure 4) is by oxygenation of Fe^2+^. Indeed, the production of soluble organic-Fe(III) complexes in these layers (e.g., Figure 2b, Figure 4, Figure 5b, and Figure 7) provides evidence for the oxidation of Fe^2+ ^in these sediments. These complexes can be rapidly produced in the presence of dissolved oxygen and natural organic ligands and are extremely stable, even in seawater, if not exposed to dissolved sulfide [30]. If dissolved oxygen penetrates deep porewaters during tidal cycles, the formation of soluble organic-Fe(III) complexes during rising and ebb tides may explain why microbial iron reduction is an important mechanism of organic carbon remineralization in creek sediments [15,62]. A back-of-the-envelope calculation illustrates the importance of this process. Assuming that sediments at SERF are at steady-state with respect to the iron cycle, the rate of microbial iron reduction measured over the first 10 cm of the sediment column [15,62] should be balanced by the oxygenation of Fe^2+^. As this process involves the oxidation of four moles of iron per mole of molecular oxygen, the rate of oxygen consumption should be a quarter of the rate of iron reduction. Microbial iron reduction rates between 70 and 116 mmole Fe m^-2 ^d^-1 ^are reported for creek sediments at SERF [15,62]. Thus, oxygen consumption rates should range between 18 and 29 mmole O_2 _m^2 ^d^-1 ^to balance the reduction of iron. Benthic fluxes of dissolved oxygen have not been measured at SERF, but total and diffusive uptake rates of oxygen in coastal sediments generally range between 10 and 75 mmole O_2 _m^2 ^d^-1 ^[11,14,63-66]. These data suggest that between 50 and 100% of the dissolved oxygen consumed could recycle iron oxides in these intertidal sediments. Therefore, while thermodynamically sulfate reduction may be a more efficient terminal electron acceptor (one mole of sulfate can oxidize two moles of carbon), iron reduction provides more energetic power to iron-reducing bacteria, and iron oxides should be very rapidly replenished near the sediment surface through tidally-induced oxygen irrigation.

## Conclusion

In this study, ex situ depth-profiling of sediment cores over several seasons, in situ depth-profiling, and time-series at several fixed depths were combined with water level measurements in monitoring wells to study the influence of tidal forcing on biogeochemical processes in the first 15 cm of intertidal salt marsh sediments. Our high spatial and temporal resolution data demonstrate that tidal forcing mostly influences the first tens of centimeters of these sediments and has different impact on creek and mud flat sediments. Creek sediments are confined environments where hydrostatic pressure gradients are high during flood and ebb tides. As a result, the porewater geochemistry changes drastically during tidal cycles. In general, dissolved sulfide seems to advect from the deep porewaters at rising tide, partly reducing iron and manganese oxides and partly precipitating FeS on its way to the surface of the sediment. However, as Fe^2+ ^is ubiquitously found in excess of dissolved sulfide in creek porewaters, another iron reduction process must prevail in these sediments. During rising and ebb tides, dissolved oxygen must penetrate the sediment, as reduced chemical species are rapidly removed from the porewaters and replaced by soluble organic-Fe(III) complexes. This hydrologic regime facilitates the recycling of iron oxides and must be responsible for the prevalence of microbial iron reduction detected by other studies in these environments. In contrast, mud flat sediments are exposed to a significantly smaller hydrostatic pressure during tidal cycles, and surface waters infiltrate the sediment. As a result, the porewater chemical composition is less variable during tides and sulfate reduction is much more prevalent during most of the year. All the evidence provided in this study indicate that advection is an important physical process affecting the biogeochemical cycling of redox sensitive elements in salt marsh sediments. Bioturbation helps increase the permeability of sediments as well as the surface area of sediments exposed to dissolved oxygen from the overlying waters, but the flushing activity of macroorganisms does probably not influence the biogeochemical cycling of elements significantly in these advective environments. This study also demonstrates that biogeochemical investigations in intertidal salt marsh sediments using conventional sediment core extractions and analyses must be carefully considered as the porewater chemical composition is highly affected by the flux of water during tidal cycles.
